# Outcomes of morbidly obese patients undergoing total hip arthroplasty with the anterior-based muscle-sparing approach

**DOI:** 10.1302/2633-1462.45.BJO-2022-0140.R2

**Published:** 2023-05-02

**Authors:** Bailey E. Shevenell, Johanna Mackenzie, Lillian Fisher, Brian McGrory, George Babikian, Adam J. Rana

**Affiliations:** 1 Maine Medical Partners Orthopedics Joint Replacement, Falmouth, Maine, USA; 2 Maine Medical Center, Portland, Maine, USA

**Keywords:** Total hip arthroplasty, ABLE™ Approach, Anterior-based muscle-sparing (ABMS) approach, Obesity, Rottinger approach, modified Watson-Jones approach, BMI, morbidly obese, total hip arthroplasties (THAs), patient-reported outcome measures (PROMs), infections, deep infections, Anaesthesia, periprosthetic fracture, comorbidities

## Abstract

**Aims:**

Obesity is associated with an increased risk of hip osteoarthritis, resulting in an increased number of total hip arthroplasties (THAs) performed annually. This study examines the peri- and postoperative outcomes of morbidly obese (MO) patients (BMI ≥ 40 kg/m^2^) compared to healthy weight (HW) patients (BMI 18.5 to < 25 kg/m^2^) who underwent a THA using the anterior-based muscle-sparing (ABMS) approach.

**Methods:**

This retrospective cohort study observes peri- and postoperative outcomes of MO and HW patients who underwent a primary, unilateral THA with the ABMS approach. Data from surgeries performed by three surgeons at a single institution was collected from January 2013 to August 2020 and analyzed using Microsoft Excel and Stata 17.0.

**Results:**

This study compares 341 MO to 1,140 HW patients. Anaesthesia, surgery duration, and length of hospital stay was significantly lower in HW patients compared to MO. There was no difference in incidence of pulmonary embolism, periprosthetic fracture, or dislocation between the two groups. The rate of infection in MO patients (1.47%) was significantly higher than HW patients (0.14%). Preoperative patient-reported outcome measures (PROMs) show a significantly higher pain level in MO patients and a significantly lower score in functional abilities. Overall, six-week and one-year postoperative data show higher levels of pain, lower levels of functional improvement, and lower satisfaction scores in the MO group.

**Conclusion:**

The comorbidities of obesity are well studied; however, the implications of THA using the ABMS approach have not been studied. Our peri- and postoperative results demonstrate significant improvements in PROMs in MO patients undergoing THA. However, the incidence of deep infection was significantly higher in this group compared with HW patients.

Cite this article: *Bone Jt Open* 2023;4(5):299–305.

## Introduction

Obesity is an epidemic in the USA as 51% of American adults were classified as obese in 2017 to 2018, a number expected to continue to rise.^1^ Due to the well-documented comorbidities of obesity in the USA, it is known that BMI is linked to hip osteoarthritis (OA).^2,3^ Obesity is a modifiable risk factor that negatively impacts cartilage of the joints through mechanical and cellular mechanisms; poor biomechanics leads to incorrect loading of weight on the joints and alters the gait, while body fat and adipokine build-causes low-grade systemic inflammation in the joints.^4^ When arthritis about the hip is advanced and a patient has failed conservative measures, a total hip arthroplasty (THA) is the definitive treatment.^5^ It is shown that obese patients have significant functional improvement and pain relief following a THA, but peri- and postoperative data have shown higher complication rates in obese patients, and even higher in individuals classified as morbidly obese (MO).^6-8^ Additionally, a recent study has shown that the obesity prevalence among primary THA patients is significantly higher than the general USA population.^9^ Studies have begun to further break out obesity into the sub-population of MO patients undergoing THA to see if this group carries with it different outcomes.^6^ Considering the projected increased demand for THAs, and the projected increase in MO patients, understanding factors that can increase optimal outcomes for these patients are critical.

Previous research on the implications of obesity on THA has been conducted using traditional and contemporary THA approaches, including the direct anterior (DA) approach and posterior approach.^10-12^ The anterior-based muscle-sparing (ABMS) approach is a lesser-known comprehensive approach that was described by Rottinger et al in 2004,^13^ using the interval between the tensor fasciae latae (TFL) posteriorly and the gluteus medius (GM) muscle anteriorly.^13-15^ This approach is a safe and effective approach that is muscle-sparing and can be performed in the supine or lateral position.^16^ Variations have been seen in the arthroplasty literature as early as 1965, and has most recently been cited as the ABLE™ (Smith & Nephew, USA) approach.^16^ To date, there are no studies that evaluate outcomes of obesity using the ABMS approach. This study aims to evaluate the peri- and postoperative outcomes of healthy weight (HW) and MO patients undergoing primary THA, stratified by BMI using the ABMS approach.

## Methods

Approval from the institutional review board was obtained. Patient identification was performed using MaineHealth’s electronic medical record (EMR) database, (EPIC Systems, USA). The study population was composed of patients who underwent a primary elective THA performed using the ABMS approach between 1 January 2013 and 31 August 2020. Patients with a preoperative diagnosis of OA, avascular necrosis, or rheumatoid arthritis were included in this study. Patients were excluded if their primary diagnosis was femoral neck fracture or impending pathological fracture. Patients who received a simultaneous bilateral THA were also excluded. There were 6,421 primary elective THA procedures using the ABMS approach during this time frame. There were 189 patients excluded based on exclusion criteria.

Patients were grouped according to their BMI. The World Health Organization defines BMI as five primary categories: underweight (BMI < 18.5 kg/m^2^); healthy weight (18.5 to < 25 kg/m^2^); overweight (25 to < 30 kg/m^2^); obese (30 to < 40 kg/m^2^); and morbidly obese ( ≥ 40 kg/m^2^).^17^ Only patients with a BMI in the HW and MO categories were included in this study. Of the 6,232 patients who met inclusion criteria, 1,440 had a HW BMI and 341 patients had a MO BMI. In total, there were 1,781 patients included in this study.

Patient demographics (sex, age, American Society of Anesthesiology (ASA) rating, BMI), primary diagnosis, anaesthesia type, anaesthesia duration, procedure duration (calculated from incision start to incision close), intraoperative estimated blood loss, blood transfusions, length of hospital stay (calculated in days from hospital admission time to hospital discharge time), and discharge disposition were retrieved from the patient database. The 30-day emergency department (ED) visits and 90-day unplanned readmissions were recorded. Postoperative complications were obtained via the EMR from a report built by an internal analyst using the Centres for Medicare and Medicaid Services codes and definition that identified both index admission complications and post-discharge complications. If a patient had the same complication twice, it was only accounted once. Additionally, guidelines from the proceedings of the international consensus on periprosthetic joint infection (PJI) was used to further classify infections.^18^ All patients with a length of stay (LOS) greater than four days had a manual chart review for added evaluation of hospital course. All postoperative complications were also confirmed through a manual chart review.

Patient-reported outcome measures (PROMs) were obtained from two databases: ORTech (Ontario, Canada), which was used by the institution for PROM data entry before 2018; and REDCap (Vanderbilt University, USA), which was used for PROM data entry after 2018. Patients completed PROM questionnaires preoperatively, six weeks postoperatively, six months postoperatively, and one year postoperatively. Patients completed the Hip Disability and Osteoarthritis Outcome Score, Joint Replacement (HOOS, JR), visual analogue scale (VAS) pain, Single Assessment Numeric Evaluation (SANE), University of California and Los Angeles (UCLA) activity score, ten-item Patient-Reported Outcomes Measurement Information System Global Health survey (PROMIS-10), and postoperative satisfaction.

### Statistical analysis

Statistical analysis of HW and MO patients was performed using Excel 2013 (Microsoft, USA) and Stata version 17.0 (StataCorp, USA). A two-tailed *t*-test assuming unequal variance was used to determine the difference in continuous variables between the two BMI groups. The chi-squared test was used to compare categorical variables. Significance was defined when the p-value was ≤ 0.05. Continuous variables were expressed as the mean and standard deviation (SD), while categorical variables are expressed as the count and percentages of the group total.

## Results

There were 1,140 HW and 341 MO patients who underwent a primary THA using the ABMS approach and were eligible for inclusion during this study period. There were several significant differences between the two groups (Table I). The mean age between groups was statistically significant (p < 0.001, two-tailed *t*-test); the mean age of HW patients was 67.3 years (29 to 97; SD 10.5), while the mean age of the MO patients was 61.3 years (35 to 83; SD 9.2). The mean BMI for the HW patients was 22.7 kg/m^2^ (18.5 to 24.9; SD 1.6), while the MO group was 44.1 kg/m^2^ (40 to 64.6; SD 4.0), and this difference was significant (p < 0.001, one-tailed *t*-test). The mean ASA and complexity scores were significantly lower (p < 0.001, two-tailed *t*-test) in the HW patients (2.0 (SD 0.5) and 3.9 (SD 1.9)) than MO (2.6 (SD 0.5) and 4.8 (SD 2.0)) patients. Preoperative VAS pain score was significantly lower (p < 0.001, two-tailed *t*-test) in the HW group than the MO group (5.3 (SD 2.2) and 6.9 (SD 2.0)), representing different baseline pain levels. The mean preoperative UCLA score demonstrated the HW group to be significantly more functional preoperatively than the MO group (4.7 (SD 1.8) and 3.1 (SD 1.3)), respectively (p < 0.001, two-tailed *t*-test).

**Table I.** Baseline characteristics of 1,781 patients undergoing primary total hip arthroplasty stratified by a healthy weight BMI and a morbidly obese BMI.

Variable	Healthy weight (BMI 18.5 < 25 kg/m^2^), n = 1440	Morbidly obese (BMI ≥ 40 kg/m^2^), n = 341	p-value*
Mean age, yrs (SD)	67.3 (10.5)	61.3 (9.2)	< 0.001
**Sex, n (%)**			0.066
Female	1,015 (70.5)	223 (65.4)	
Male	425 (29.5)	118 (34.6)	
Mean BMI, kg/m^2^ (SD)	22.7 (1.6)	44.1 (4.0)	< 0.001
Mean ASA classification	2.0 (0.5)	2.6 (0.5)	< 0.001
Mean complexity (SD)	3.9 (1.9)	4.8 (2.0)	< 0.001
Mean preoperative VAS pain score (SD)	5.3 (2.2)	6.9 (2.0)	< 0.001
Mean preoperative UCLA score (SD)	4.7 (1.8)	3.1 (1.3)	< 0.001

* Variables presented as counts and percents were analyzed using chi-squared test. Variables presented as mean and SD were analyzed using one- or two-tailed *t*-tests.

ASA, American Society of Anesthesiology; SD, standard deviation; UCLA, University of California and Los Angeles; VAS, visual analogue scale.

Both the HW and MO group had similar anesthetic treatment (Table II); 97.01% (n = 1,398) of HW patients received general anaesthesia and 2.92% (n = 42) received spinal anaesthesia, while 97.95% (n = 334) of MO patients received general anaesthesia and 2.05% (n = 7) received spinal anaesthesia (p = 0.061). There was a significant difference in anaesthesia duration between the two groups (p < 0.001) with the HW patients undergoing a mean of 103.9 minutes (SD 19.9) of anaesthesia and the MO group undergoing a mean of 119.3 minutes (SD 20.4) of anaesthesia (Table II). The length of surgery significantly differed between the two groups (p < 0.001), with the mean surgery duration for HW patients being 62.0 minutes (SD 17.9) and 73.5 minutes (SD 16.6) for the MO group (Table II). The mean estimated blood loss (EBL) for patients in the HW group was significantly less (p < 0.001) than MO patients, with blood loss at 208.9 ml (SD 71.4) and 248.1 ml (SD 88.7), respectively. Regardless of the difference in blood loss, there was no significant difference in blood transfusion rates within seven days of surgery between the two groups (p = 0.828). The mean LOS was also significantly higher (p < 0.001) in the MO group than the HW group, with LOS mean at 1.50 days (SD 0.79) for MO patients, and 1.36 days (SD 0.63) for HW patients (Table II).

**Table II.** Surgical and perioperative data.

Variable	Healthy weight (BMI 18.5 < 25 kg/m^2^), n = 1440	Morbidly obese (BMI ≥ 40 kg/m^2^), n = 341	p-value*
**Anaesthesia, n (%)**			0.061
General	1398 (97.01)	334 (97.95)	
Spinal	42 (2.92)	7 (2.05)	
Mean anaesthesia duration, mins (SD)	103.9 (19.9)	119.3 (20.4)	< 0.001
Mean length of surgery, mins (SD)	62.0 (17.9)	73.5 (16.6)	< 0.001
Mean estimated blood loss, ml (SD)	208.9 (71.4)	248.1 (88.7)	< 0.001
**Blood transfusion, n (%)**			0.828
Yes	11 (0.76)	3 (0.88)	
No	1429 (99.2)	338 (99.1)	
Mean length of stay, days (SD)	1.36 (0.63)	1.50 (0.79)	0.001

* Variables presented as counts and percents were analyzed using chi-squared test. Variables presented as mean and SD were analyzed using one- or two-tailed *t*-tests.

Postoperative complications were experienced by eight patients in the HW group (0.56%), and eight patients in the MO group (2.35%). Instances of infection occurred in 0.14% of HW patients, and 1.47% of MO patients, representing a significantly higher rate (p < 0.001) in the MO population. The infections include both superficial and deep infections. The infections in the MO group represents four (80%) superficial wound infections and one (20%) deep infection, while the infections in the HW group represents one (50%) superficial wound infection and one (50%) deep infection. Other complications of note include fracture and dislocation, representing 0.35% and 0.07% of the HW population, and 0.88% and 0% of the MO population, respectively (Table III).

**Table III.** Postoperative data.

Complication	Healthy weight (BMI 18.5 < 25 kg/m^2^), n = 1440	Morbidly obese (BMI ≥ 40 kg/m^2^), n = 341	p-value*
**Fracture, n (%)**			0.186
Yes	5 (0.35)	3 (0.88)	
No	1,434 (99.65)	338 (99.12)	
**Infection, n (%)**			< 0.001
Yes	2 (0.14)	5 (1.47)	
No	1,438 (99.86)	336 (98.53)	
**PE/DVT, n (%)**			
Yes	0 (0.00)	0 (0.00)	
No	1,440 (100.00)	341 (100.00)	
**Dislocation, n (%)**			0.626
Yes	1 (0.07)	0 (0.00)	
No	1,439 (99.93)	341 (100.00)	
**ED visit within 30 days, n (%)**			0.848
Yes	32 (2.22)	7 (2.05)	
No	1,408 (97.78)	334 (97.95)	
**Readmission within 90 days, n (%)**			0.312
Yes	40 (2.78)	13 (3.81)	
No	1,400 (97.22)	328 (96.19)	
**Discharge disposition, n (%)**			0.449
Home or self care	1,327 (92.15)	310 (90.91)	
Rehab or skilled nursing facility	113 (7.85)	31 (9.09)	

* Variables presented as counts and percents were analyzed using chi-squared test. Variables presented as mean and SD were analyzed using one- or two-tailed *t*-tests.

DVT, deep vein thrombosis; ED, emergency department; PE, pulmonary embolism.

PROM questionnaires were sent out preoperatively (within one month prior to surgery) and postoperatively at different time points (six weeks and one year) to observe trends in functional ability, pain, and patient satisfaction before and after surgery. These voluntary questionnaires ranged in participation and completion rates due to the nature and modality of the questionnaires. Of the total population, there was a preoperative PROM completion rate of 70.8% (70.4% of the total HW cohort and 72.9% of the total MO cohort), a completion rate of 57.0% for six-week postoperative PROM (56.8% of HW patients and 57.7% of MO patients), and a preoperative completion rate of 43.7% for the one-year postoperative PROM (42.7% of the HW cohort and 48.0% of the MO cohort). Preoperative PROM questionnaire data demonstrated that the MO patients had higher pain scores and reduced activity levels at baseline than the HW cohort; both of which were significant differences (Table I). The six-week and one-year postoperative PROM results showed the same trends in higher pain levels and lower activity in MO patients. Satisfaction data taken at six weeks shows that the MO group was more satisfied with their pain relief, functional improvement, procedure expectations, and surgeon experience (Table IV).

**Table IV.** Patient-reported outcome measures and clinical outcomes.

Variable	Healthy weight (BMI 18.5 < 25 kg/m^2^), n = 1440	Morbidly obese (BMI ≥ 40 kg/m^2^), n = 341	p-value*
**Mean six-week postoperative (SD)**			
Pain (n = 987)	1.41 (1.6)	1.51 (1.8)	0.219
UCLA (n = 983)	5.03 (1.4)	4.52 (1.2)	< 0.001
HOOS, JR (n = 847)	77.67 (13.0)	75.35 (13.9)	0.012
PROMIS physical (n = 971)	46.50 (5.4)	42.32 (5.0)	< 0.001
PROMIS mental (n = 971)	52.55 (7.1)	49.15 (6.5)	< 0.001
SANE (n = 905)	76.96 (17.6)	79.95 (16.1)	0.209
**Mean six-week satisfaction (SD)**			
Pain relief (n = 826)	8.92 (1.6)	9.14 (1.5)	0.061
Functional improvement (n = 818)	8.62 (1.7)	8.89 (1.5)	0.033
Procedure met expectations (n = 810)	9.04 (1.6)	9.27 (1.5)	0.036
Surgeon (n = 835)	9.78 (0.7)	9.85 (0.7)	0.097
**Mean one-year postoperative (SD)**			
Pain (n = 531)	0.67 (1.3)	0.98 1(.8)	0.041
UCLA (n = 525)	6.45 (1.9)	6.02 (1.7)	0.009
HOOS, JR (n = 447)	89.75 (13.7)	85.27 (13.5)	0.002
PROMIS physical (n = 628)	48.91 (6.4)	44.51 (5.7)	< 0.001
PROMIS mental (n = 628)	52.90 (7.1)	50.23 (6.8)	< 0.001
SANE (n = 469)	92.09 (12.6)	89.72 (16.7)	0.096

* Variables presented as counts and percents were analyzed using chi-squared test. Variables presented as mean and SD were analyzed using one- or two-tailed *t*-tests.

HOOS, JR, Hip Disability and Osteoarthritis Outcome Score, Joint Replacement; PROMIS, Patient-Reported Outcomes Measurement Information System; SANE, Single Assessment Numeric Evaluation; SD, standard deviation; UCLA, University of California and Los Angeles.

## Discussion

This study was the first to evaluate perioperative and short-term postoperative results in patients that underwent a primary THA using the ABMS approach stratified by BMI. According to a survey from the American Association of Hip and Knee Surgeons, more than 50% of surgeons are shifting towards an anterior-based approach, such ABMS or DA, from a more traditional posterior or lateral approach.^19^ To date, there is limited data on the ABMS approach; the majority of studies have primarily evaluated the posterior, lateral, or DA approach.^20,21^ Additionally, studies have shown that the DA approach is associated with higher rate of infection in some patients.^12,16^ Due to the prevalence of MO, the demand for THA will follow to alleviate musculoskeletal disease related to obesity and it is imperative to observe new techniques to improve outcomes in an already at-risk population.^9,22^

In a meta-analysis by Onggo et al,^6^ when compared to non-obese patients, obese individuals were at a significantly greater risk of all complications, deep and superficial infections, reoperations, revisions, dislocations, and readmissions. In a sub-group analysis of MO patients compared to non-obese patients, these risks were even greater. Various studies have shown that increased operation time is a risk factor for PJI in THA, as each 20 minute operative time increases the risk by about 25%.^23^ Our data, using the ABMS approach, is different than these findings in that we did not observe an increased incidence of dislocations, periprosthetic fracture, reoperations, or readmissions. However, we did note an increased incidence of infection in the MO group which is consistent with these studies (Table III). According to a large review by Moretti et al,^24^ the incidence of infection after primary THA in most large studies report to be 0.2 to 1.2%, although approach was largely not evaluated. More specifically, the incidence of infection in a recent retrospective study of primary THA comparing the ABMS and DA approaches reports a 5.6% complication rate in the ABMS approach, with infection being the most common at 2.1%.^21^ The incidence of infection in our HW population (0.14%) is less than these averages. The complication rates in our study, including revision surgeries and dislocations, are low and comparable to other surgical approaches to THA.^6,12,25-27^

It was found in this study that the time under anaesthesia, total surgical time, and LOS are all significantly higher in MO patients. These results are widely consistent with many previous studies that stratify BMI using an approach other than the ABMS approach.^6,12,26^ The length of surgery is reflected by the increased complexity due to the size of the MO patient, and is also widely concordant with other studies comparing perioperative parameters of THA using other approaches including the DA and posterior approaches.^28^ The EBL was also significantly higher in MO versus HW patients; however, the instances of blood transfusions within seven days postoperatively did not represent any significant differences between groups. These trends were also similar to other studies using other approaches cited in the literature.^26,28^

Our demographic data shows that our two cohorts represent significantly different populations in all categories (Table I). The complexity and ASA scores describe the MO group to have significantly more comorbidities than the heathy weight population. In addition, in comparison to the HW patients, MO patients are significantly younger at the time of surgery, suggesting an earlier onset and accelerated progression of OA.^6,29,30^ Previous research has shown that younger individuals have a lower risk of periprosthetic fracture and revision due to increased bone density, which might have been reflected in this cohort.^31^ It is known that every two-unit (5 kg) increase in BMI increases the chance of developing degenerative joint disease by 36%.^32^ This trend toward younger patients is increasingly concerning due to the increased risk of revision and perioperative complications in this patient population.^6^

This retrospective study has several limitations. A prospective, randomized controlled trial would be the gold standard method to compare patient outcomes by surgical approach. The retrospective, non-randomized design could be subject to surgeon preference which could influence results; however, all surgeons use this approach in > 99% of their primary THAs. This study is a smaller subset of patients from a large review of 6,251 THA procedures performed with the ABMS approach. This previous study was performed to summarize our institutions’ observations with this approach, and to observe the overall efficacy compared to published literature. Using a subset of these patients for this analysis therefore provided readily available data to observe trends in BMI categories. In order to determine definitive conclusions between surgical approaches, consideration of a prospective study protocol with matched sample sizes should be used. Additionally, this study would be strengthened if our hospital had a higher volume of patients that had undergone a THA with an approach other than the ABMS approach, to perform a comparison of approaches within the same surgeons and institution. Regardless, the high-volume of THA procedures that were performed in our previous study emphasizes the experience with the ABMS approach.

Another limitation of this study is that our results come from one single EMR. This restricts pulled information to being only from the ten hospitals in the state within our institution’s network. Due to this, we are limited to readmissions and ED visits documented by one of these hospitals. If a patient sought medical care at a hospital that is not within our network, it was not included as a postoperative complication in our study. Similarly, our postoperative outcomes were limited to hospital data recording during patients’ inpatient stay, excluding the ability to extrapolate other comparative outcomes such as gait. Patient outcomes were also only followed on a short-term (90 days) postoperative basis, excluding PROM data, which was followed up to one year postoperatively. Observing outcomes such as revision THA could be a notable comparison if patients were followed further.

The small sample size of MO patients is another limitation due to the fact that our patients are only representative of a single institution. Propensity score matching patient cohorts would strengthen this study. Our method of collecting PROMs is by voluntary questionnaire. This presents another limitation because this is not a reflection of the entire group, but rather only patients that choose to complete them. Additionally, as our preoperative optimization programme emphasizes a BMI less than 40 kg/m^2^, the MO patients studied may be a select subset and not a true representation of the MO population as a whole.

**Take home message**

- There is currently no data on how the anterior-based muscle-sparing (ABMS) approach compares to other total hip arthroplasty approaches when stratified by BMI.

- This study aids to fill the gap of knowledge of the ABMS approach in terms of morbidly obese patients, and demonstrates its efficacy in managing this patient population.
